# The combinatorial binding syntax of transcription factors in forebrain-specific enhancers

**DOI:** 10.1242/bio.061751

**Published:** 2025-02-19

**Authors:** Fatima Batool, Huma Shireen, Muhammad Faizan Malik, Muhammad Abrar, Amir Ali Abbasi

**Affiliations:** National Center for Bioinformatics, Program of Comparative and Evolutionary Genomics, Faculty of Biological Sciences, Quaid-i-Azam University, Islamabad 45320, Pakistan

**Keywords:** Forebrain, Enhancers, Transcription factors, Gene regulation, Epigenetics, Zebrafish

## Abstract

Tissue-specific gene regulation in mammals involves the coordinated binding of multiple transcription factors (TFs). Using the forebrain as a model, we investigated the syntax of TF occupancy to determine tissue-specific enhancer regions. We analyzed forebrain-exclusive enhancers from the VISTA Enhancer Browser and a curated set of 23 TFs relevant to forebrain development and disease. Our findings revealed multiple distinct patterns of combinatorial TF binding, with the HES5-FOXP2-GATA3 triad being the most frequent in forebrain-specific enhancers. This syntactic structure was detected in 2614 enhancers from a genome-wide catalog of 25,000 predicted human forebrain enhancers. Notably, this catalog represents a computationally predicted dataset, distinct from the *in vivo* validated set of enhancers obtained from the VISTA Enhancer Browser. The shortlisted 2614 enhancers were further analyzed using genome-wide epigenetic data and evaluated for evolutionary conservation and disease relevance. Our findings highlight the value of these 2614 enhancers in forebrain-specific gene regulation and provide a framework for discovering tissue-specific enhancers, enhancing the understanding of enhancer function.

## Teaser

**Summary:** The co-occurrence patterns of tissue-relevant transcription factor binding motifs can serve as a general framework for tissue-specific enhancer discovery.

## INTRODUCTION

The precise patterns of gene expression encoded within the genome determine everything from the stereotypic development of an embryo to the species-specific complex body plans of an animal (Davidson, 2010). These gene expression programs are, in turn, highly regulated by large and interconnected networks of *cis-*regulatory elements (Oliveri et al., 2008), which include core and proximal promoter regions near the transcription start site (TSS) and distant elements like enhancers (Levine, 2010; Lenhard et al., 2012). Enhancers, typically short stretches of DNA with binding sites for transcription factors (TFs), serve as platforms to recruit TFs and regulate gene expression (Ganten and Ruckpaul, 2006). Enhancers are classified as *cis*-acting sequences of DNA capable of increasing gene transcription levels (Pennacchio et al., 2013). They function independently of their orientation and can influence transcription from thousands of base pairs upstream or downstream of the transcription initiation site (Davidson and Erwin, 2006; Pennacchio et al., 2013).

The study of enhancers has gained significant attention since their first discovery over 40 years ago in simian virus 40 (SV40) (Rio et al., 1980; Banerji et al., 1981). Researchers have sought to understand how these short DNA sequences enable complex patterns of spatiotemporal gene regulation and how TFs interpret the genome, to ensure the activation of the right sequences at each developmental stage (Banerji et al., 1981). TFs typically act in a combinatorial manner rather than in isolation (Guo and Gifford, 2017).

The temporal control of gene regulation is defined not merely by the presence or absence of specific TFs but by the timing of their DNA binding (Jakobsen et al., 2007; Sandmann et al., 2007). This binding is influenced by context-dependent factors, including relative affinity and the number of available binding sites (Gaudet and Mango, 2002; Sandmann et al., 2006). Cooperative TFs occupancy is determined by the motif architecture of *cis*-regulatory elements, where specific motif arrangements offer insights into enhancer function (Zinzen et al., 2009; Parveen et al., 2013; Ali et al., 2016). Motif architecture refers to motif composition (the presence of specific TF binding sites) and motif grammar (the relative order, orientation, and spacing of these binding sites within an enhancer) (Spitz and Furlong, 2012). A study using massively parallel experiments revealed that heterotypic enhancers, with diverse TF binding sites, are potent drivers of gene expression compared to homotypic enhancers (Smith et al., 2013). Specific motif combinations associated with strong reporter expression are prevalent in heterotypic enhancers (Smith et al., 2013). Numerous studies underscore the significance of context-dependent co-occupancy of TFs in the spatiotemporal gene regulatory activity of enhancers (Narlikar et al., 2010; Brody et al., 2012).

Despite the discovery of *cis*-acting gene regulatory elements decades ago and recent advances in identifying tissue-specific enhancers using genetic, evolutionary, and biochemical approaches, our understanding of the principles governing enhancer tissue specificity remains elusive (Pennacchio et al., 2007). The commonly accepted view is that enhancer functionality arises from the non-stereotypic binding of multiple distinct TFs, allowing for adaptable and context-dependent regulation (Guo and Gifford, 2017). Two models describe the cooperative binding of TFs to tissue-specific enhancers: (i) the ‘enhanceosome’ model, requiring rigid and precise positioning of TF-binding motifs, and (ii) the ‘billboard’ model, allowing flexible positioning and spacing of TF motifs (Merika and Thanos, 2001; Arnosti and Kulkarni, 2005).

Understanding tissue-specific TF occupancy would deepen our molecular and biochemical knowledge of enhancer functionality and aid in computational strategies for tissue-specific enhancer discovery. This study aims to elucidate the principles behind the cooperative interaction of TFs that confer tissue-specific gene expression to mammalian non-coding genomic regions. We evaluated the binding patterns of a curated set of forebrain-relevant TFs on a dataset of forebrain-specific human enhancers (FSHEs) from the VISTA Enhancer Browser (Visel et al., 2006). The HES5-FOXP2-GATA3 triad, where FOXP2 binding site is positioned between HES5 and GATA3, showed the most pronounced binding pattern. Binding site spacing analysis revealed that forebrain-relevant syntactic association among HES5-FOXP2-GATA3 is constrained in terms of the order of factors but remains flexible regarding spacing. Thus, our findings position the proposed tissue-specific genomic occupancy of TFs between the rigid enhanceosome and flexible billboard models.

## RESULTS AND DISCUSSION

### Discovery of tissue-specific TFs binding motif grammar in experimentally validated human forebrain enhancers

Tissue-specific transcriptional output of enhancers is governed by a set of grammatical rules composed of the linear arrangement of transcription factor binding sites (TFBSs), their number, type, affinity, order, spacing and orientation (Weingarten-Gabbay and Segal, 2014; Barolo, 2016; Jindal and Farley, 2021). Transcription factors (TFs) are known to collaborate through specific co-binding at *cis*-regulatory modules (CRMs) to mediate precise gene expression (Lebrecht et al., 2005; Mirny, 2010; Lambert et al., 2018). Identifying tissue/cellular specific, context dependent co-occupancy of TFs is thus critical for deciphering the transcriptional regulatory code of tissue-specific gene expression (Wang et al., 2018).

To define the core transcriptional regulatory code (grammar) specific to human/mammalian forebrain, we utilized a functionally validated dataset of forebrain-specific human enhancers (FSHEs) from the VISTA Enhancer Browser (Visel et al., 2006), alongside a set of 23 key TFs identified as fundamental regulators of forebrain development (Shireen et al., 2025) (Tables S1,S2). The enhancers selected from the VISTA Enhancer Browser are known for their robust and reproducible spatial expression patterns, demonstrating strong enhancer activity exclusively in the forebrain of transgenic mice. These 100 FSHEs were specifically chosen to represent a broader spectrum of the forebrain enhancer landscape, providing a foundation for investigating the combinatorial binding patterns of TFs. The catalog of 23 forebrain-relevant TFs was constructed through the following steps:

- A literature-based survey to evaluate their endogenous expression in forebrain tissues, their role in forebrain development, and associated diseases.
- Identification of evolutionarily conserved overrepresented binding motifs in *in-vivo* characterized forebrain-specific enhancers from the VISTA Enhancer Browser (Visel et al., 2006).
- Enrichment analysis of their binding motifs in DNase I hypersensitive sites derived from forebrain tissue samples, specifically ENCODE (Encyclopedia of DNA Elements) derived Cerebrum_Frontal_OC (frontal cerebrum) and Frontal_Cortex_OC (ventromedial prefrontal cortex) (Feingold et al., 2004).
- Statistical evaluation of their tendency to co-bind to forebrain-specific enhancers.

A flowchart detailing various steps that led to the identification of this curated set of 23 TFs highly relevant to the functionality of forebrain enhancers is summarized in Fig. S1, which is adapted from Shireen et al. (Shireen et al., 2025).

**Fig. 1.**
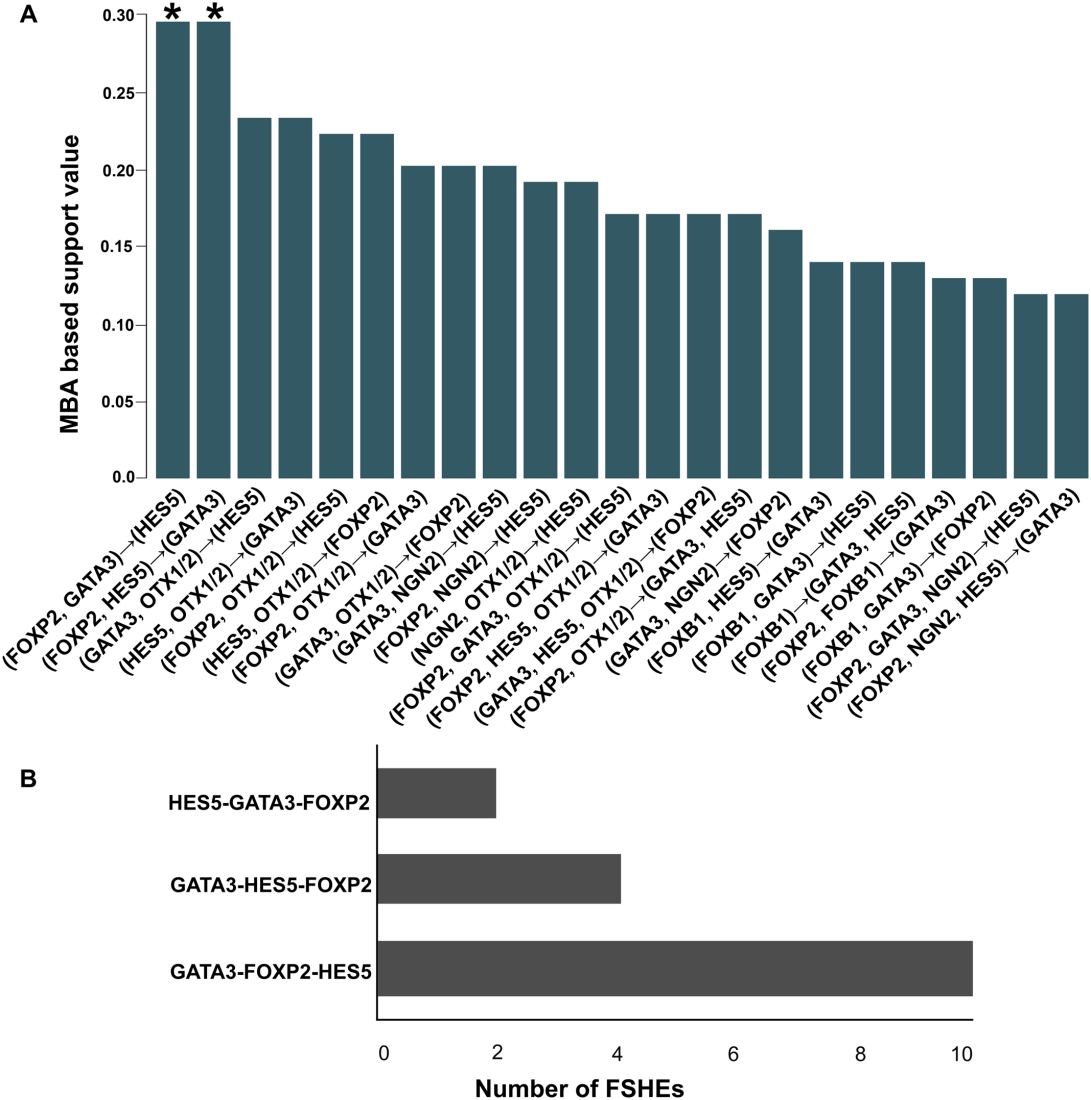
**Transcription factors binding motif combinations identified in human forebrain-specific enhancers.** (A) The vertical bar graph depicts the MBA-based support values of association rules observed for significant combinations among 23 TF binding motifs, identified by mining the dataset of 100 FSHEs (positive control) from the VISTA Enhancer Browser (https://enhancer.lbl.gov) (Table S4). The X-axis represents the association rules of motif combinations (involving at least three distinct TF binding motifs), while the Y-axis shows the support value (relative frequency) of these motif combinations in FSHEs (positive control). The arrows under each bar represent the TFs association rule derived from the market basket association analysis. For example, the rule (FOXP2, GATA3)→(HES5) indicates that where binding motifs for FOXP2 and GATA3 are present, there is often also a motif for HES5. The binding motif combinations of TFs HES5, FOXP2, and GATA3 appears most frequently in FSHEs with highest association rule support (0.277) indicating the co-occurrence of these three TFs in ∼28% of enhancers, represented as bar plots marked with asterisk (*). (B) The horizontal bar graph shows the relative frequency of motif order-based arrangements of HES5, FOXP2, and GATA3 among the 16/100 FSHEs where their binding motifs are positioned directly adjacent to each other with no intervening TFBSs (Table S1). The binding pattern where FOXP2 is positioned between HES5 and GATA3 occurs most frequently (10/16, 62.5%). This is more common than the other two combinations where HES5 (4/16, 25%; Fisher's exact test yielded a test statistic of 0.0732, indicating significance at *P*<0.10) or GATA3 (2/16, 12.5%; Fisher's exact test yielded a test statistic of 0.0091, indicating significance at *P*<0.05) is positioned between the other two factors. TFs, transcription factors; FSHEs, forebrain-specific human enhancers; NCNCSs, non-coding, non-conserved sequences.

To identify the forebrain-specific combinatorial binding syntax of transcription factors, we searched for the binding sites of these 23 forebrain-relevant TFs, using a customized Perl Script (source code available at GitHub repository: https://github.com/HumaShireen/TFBSMA) in a positive control dataset of 100 FSHEs (Table S1) and negative control data set of 100 non-coding non-conserved sequences (NCNCSs) (Table S3). Specific associations among the 23 forebrain-relevant TFs were mined in FSHEs (positive control) and NCNCSs (negative control) using market basket analysis (MBA) (Morgan et al., 2007; Raeder and Chawla, 2011; Bentsen et al., 2022) (source code at: https://github.com/Fatiima-Batool/Enhancer-Combinatorial-Syntax/tree/main/MBA_Python). This association rule-based mining in positive control dataset of 100 FSHEs identified several association rules with significant support, confidence and lift >1, indicating the co-occurrence of TF binding motifs in pairs as well as in combinations of three or four distinct TF binding motifs (Fig. 1A; Table S4). To define the minimal forebrain-specific regulatory code, we looked for the association rules involving at least three distinct TFs binding motifs with the highest support and confidence (Table S4; Fig. 1A). The strongest association rule (support: 0.277, confidence: 0.77, lift: 1.12) was observed for (FOXP2, GATA3)→(HES5), which indicates ‘where there is a motif for FOXP2 and GATA3, there is often also a HES5 binding motif’ suggesting this TF triad co-occurs in ∼28% of FSHEs with 77% confidence (positive control). (Table S4; Fig. 1A). Notably in NCNCSs (negative control), only pair-wise TF binding motif associations rules with relatively lower support and confidence were identified (Table S4). Detailed metrics for support, confidence, and lift for each association rule observed in the FSHEs and NCNCSs datasets are presented in Table S4.

Manual inspection of the binding matrix of 23 TFs across the positive control dataset (100 FSHEs, Table S1) revealed 16 FSHEs in which the over-represented triad of HES5, FOXP2, and GATA3 (MBA-based support: 0.277 and confidence: 0.77) had binding motifs positioned directly adjacent to one another, with no intervening TF motifs. (Table S1). Among these 16 FSHEs, the most frequent pattern, in which the FOXP2 binding motif was positioned between HES5 and GATA3 (HES5-FOXP2-GATA3) was observed in ten FSHEs (10/16, 62.5%) (Fig. 1B). In contrast, alternative motif arrangements with either GATA3 or HES5 binding motifs in the central position, i.e. HES5-GATA3-FOXP2 and GATA3-HES5-FOXP2, were detected in 2/16 (12.5%; Fisher's exact test yielded a test statistic of 0.0091, indicating significance at *P*<0.05) and 4/16 (25%; Fisher's exact test yielded a test statistic of 0.0732, indicating significance at *P*<0.10) FSHEs, respectively (Fig. 1B). Thus, the unique arrangement in which the FOXP2 binding site is centered between HES5 and GATA3 (HES5-FOXP2-GATA3), along with the combined occurrence of these three factors, was established as the syntactic pattern for forebrain enhancers (Fig. 1B). For these ten FSHEs, the spacing between adjacent TF binding sites for TF triad HES5-FOXP2-GATA3 was evaluated (Table S5). The spacing between GATA3-FOXP2 sites ranged from 35 bp in hs240 to 504 bp in hs957, with an average distance of 189 bp±150 bp (Table S5). In contrast, the spacing between HES5-FOXP2 sites ranged from 43 bp in hs123 to 813 bp in hs1526, with an average distance of 305 bp±214 bp (Table S5). These findings indicate flexibility in binding site spacing (Table S5). Two general models describe how TF binding sites work together in enhancers: the ‘enhanceosome’, which requires rigid and precise positioning of TF-binding motifs for high cooperativity, and the ‘billboard’ model, which allows more flexible positioning and spacing of motifs (Thanos and Maniatis, 1995; Merika and Thanos, 2001; Kulkarni and Arnosti, 2003; Arnosti and Kulkarni, 2005). These two models represent two extremes of the spectrum. In reality, there is no absolute classification of enhanceosome and billboard enhancers as there is no critical threshold of cooperativity or inflexibility measured or defined (Arnosti and Kulkarni, 2005). Most enhancers are likely to incorporate the aspects of both models (Jindal and Farley, 2021). Our proposed model of forebrain-specific syntax comprising TF triad HES5-FOXP2-GATA3 shows a constrained arrangement of factors on a typical forebrain enhancer but remains flexible regarding the spacing among them (Fig. 1B; Table S5). This places our model within the spectrum between the rigid enhanceosome and the flexible billboard organization (Table S5).

**Fig. 2.**
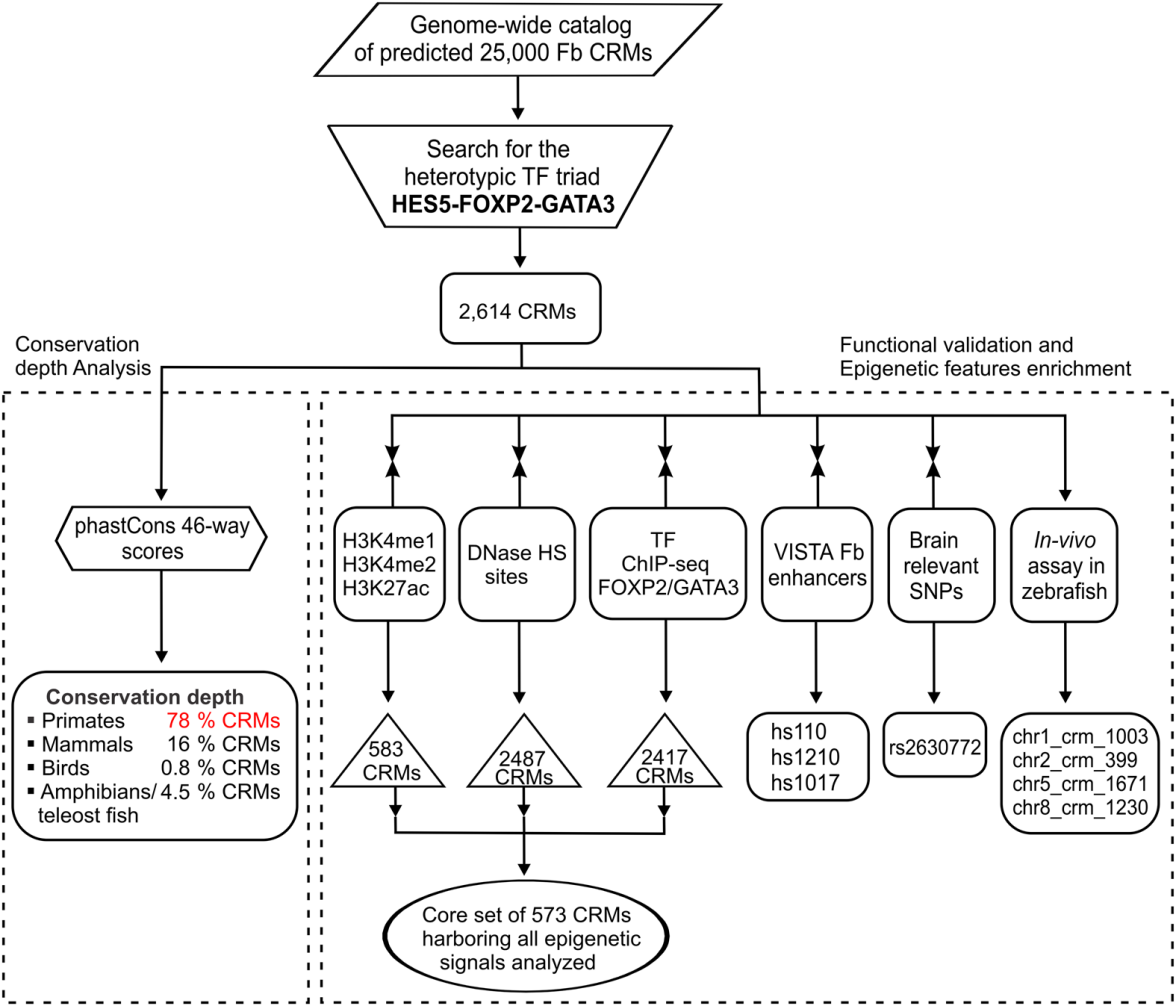
**Identification and validation scheme for human forebrain *cis*-regulatory modules with the triad pattern HES5-FOXP2-GATA3.** This flow chart outlines the process of identifying and validating forebrain-specific CRMs containing the TF triad pattern, where FOXP2 is positioned between HES5 and GATA3 (HES5-FOXP2-GATA3). Among a genome-wide catalog of approximately 25,000 predicted human forebrain CRMs available publicly at Dryad (https://datadryad.org/stash/share/LpDZxNHctzQGPr8AmBHwT8FAQOTkQohet7nBO2DlNe0), the TF triad pattern HES5-FOXP2-GATA3 was successfully detected in 2614 CRMs. (Left) An evolutionary conservation depth analysis using phastCons 46way scores (http://hgdownload.cse.ucsc.edu/goldenpath/hg19/phastCons46way/), revealed that a large majority (2051/2614; ∼78%) of the CRMs that harbor the TF triad pattern are conserved among primates only (phastCons score:<0.2). Intriguingly, only a small proportion of these 2614 forebrain CRMs were found to be conserved outside mammals (Table S7). (Right) To further evaluate their functional relevance, these 2614 CRMs that harbor the TF triad pattern HES5-FOXP2-GATA3 were intersected with various epigenetic hallmarks of mammalian *cis*-regulatory modules, such as human embryonic cerebral cortex-based activating histone modification marks H3K4me2 and H3K27ac from the Gene Expression Omnibus-NCBI, and human brain specific H3K4me1 from ENCODE (Encyclopedia of DNA Elements; https://www.encodeproject.org/) as well as DNase I hypersensitive sites (DNase HS sites) from human brain cell lines/primary tissues derived from ENCODE and Gene Expression Omnibus-NCBI (Tables S8-S10). They were also evaluated for their *in vitro* potential to bind to TFs GATA3 and FOXP2 (Tables S11,S12). This resulted in the shortlisting of a core dataset of 573 out of 2614 forebrain CRMs that are enriched for brain-specific activating histone modification marks, epigenetic marks for TFs GATA3 and FOXP2, and DNase HS sites (|Table S15). These 2614 CRMs were further validated for their functional relevance through intersection with GWAS-derived brain-related disease SNP data (Table S14), and *in vivo* validated forebrain-relevant enhancers from the VISTA Enhancer Browser (Table S13). A subset of these 2614 CRMs, with varying epigenetic and conservation features (chr2_crm_399, chr1_crm_1003, chr5_crm_1671 and chr8_crm_1230; Table S16), were subjected to zebrafish-based *in vivo* transgenic assays (Fig. 4 and Fig. S2). CRM, *cis*-regulatory module; Fb, forebrain; DNase HS, DNase hypersensitive sites; H3K4me1, histone H3 lysine 4 mono-methylation; H3K4me2, histone H3 lysine 4 di-methylation; H3K27ac, histone H3 lysine 27 acetylation; SNPs, single nucleotide polymorphisms; ENCODE, Encyclopedia of DNA Elements.

Genetic mutations in FOXP2, a Winged-Helix transcription factor, is associated with neurodevelopmental diseases in humans, leading to severe speech and language disorders (Graham and Fisher, 2013). Similarly, experiments with model animals, such as songbirds and mice, have shown that disruption of Foxp2 functionality results in deficits in song learning and ultrasonic vocalization, respectively (Shu et al., 2005). In line with these observations, FOXP2 is extensively expressed in the striatum of rodents and humans, a cluster of neurons in the subcortical region of the forebrain that coordinates multiple aspects of cognition, including the efficiency and fluidity of language (Jacquemot and Bachoud-Lévi, 2021). HES5, a basic Helix-Loop-Helix protein, is highly expressed in neural stem cells and regulates mammalian neocortical development, embryonically in the rostral part of the forebrain (Kageyama et al., 2008; Bansod et al., 2017). Mutations in the activating transcription factor GATA3 have been associated with neuronal diseases, cognitive disability, and craniofacial phenotypes (Muroya et al., 2001). Additionally, targeted disruption of GATA3 has been reported to cause severe abnormalities in mice (Pandolfi et al., 1995). Supporting these genetic findings, GATA3 is broadly expressed in the mouse central nervous system during development (Nardelli et al., 1999). Taken together, the endogenous expression, disease relevance, and significant developmental roles of these TFs, suggest that their motif co-occurrence might determine the forebrain specificity of mammalian enhancers.

### Genome-wide shortlisting of human forebrain-specific CRMs using heterotypic TF triad: HES5-FOXP2-GATA3

Numerous studies have analyzed the grammar pattern of regulatory regions. For instance, Guo et al. explored the pairwise co-binding relationships of 14 TFs in mouse embryonic stem cells using an integrative computational program called GEM (genome-wide event finding and motif discovery) (Guo et al., 2012). Another study employed a machine learning algorithm, Random Forest, to support the hypothesis that motif combination patterns are cell type-specific (Wang et al., 2018).

Recognizing that TF combinations underpin the specificity of eukaryotic gene expression regulation, we identified a significant forebrain-specific motif grammar (HES5-FOXP2-GATA3) from experimentally confirmed FSHEs in the VISTA Enhancer Browser (Visel et al., 2006). The enrichment of the HES5-FOXP2-GATA3 combination in FSHEs suggests that these TFs collaborate in regulating human forebrain-specific genes. Utilizing this forebrain-specific regulatory motif (HES5-FOXP2-GATA3), we refined an existing catalog of approximately 25,000 predicted human forebrain-specific CRMs (*cis*-regulatory modules), which were identified through computational heterotypic clustering of TFBSs with a spacer distance≤250 bp (Shireen et al., 2025). This catalog is accessible on the DATADRYAD repository platform (https://datadryad.org/stash/share/LpDZxNHctzQGPr8AmBHwT8FAQOTkQohet7nBO2DlNe0). We thoroughly examined the catalog for the characterized ordered combination of TFs (HES5-FOXP2-GATA3) using a customized Python script (https://github.com/Fatiima-Batool/Enhancer-Combinatorial-Syntax/blob/main/Code.py). Among these previously predicted 25,000 human forebrain enhancers (also termed as forebrain CRMs), our analysis identified a discrete set of 2614 CRMs exhibiting co-occurrence of HES5, FOXP2, and GATA3 binding motifs, with a specific ordered binding arrangement where FOXP2 site is positioned between HES5 and GATA3 sites (Fig. 2; Table S6). By identifying the heterotypic TF triad HES5-FOXP2-GATA3 within human forebrain-specific enhancers, we have highlighted a critical regulatory mechanism that governs gene expression in the mammalian forebrain. Furthermore, this refined catalog offers a valuable resource for further exploration of the roles of *cis*-acting gene regulatory elements in forebrain development, disease, and evolution.

### The forebrain-relevant TF triad pattern HES5-FOXP2-GATA3 may have evolved recently in mammalian evolution

Previous studies suggest that enhancers associated with the regulation of fundamental biological processes, such as embryonic development are evolutionarily conserved (Pennacchio et al., 2006; Lindblad-Toh et al., 2011). However, recent research comparing key mammalian genome sequences indicates that enhancers may change rapidly during evolution (Cotney et al., 2013; Villar et al., 2015). These recently evolved enhancers have been linked to significant phenotypic outcomes and evolutionary differences among various organisms (Ludwig et al., 2005; Pollard et al., 2006; Mclean et al., 2011; Arnold et al., 2014; Khatoon et al., 2023). For instance, gain of lineage-specific regulatory functions in human embryonic limb enhancers compared to non-human primates and mice has been noticed (Prabhakar et al., 2008; Cotney et al., 2013). Furthermore, studies suggest that human-specific mutations in enhancers led to functional gains in embryonic brain development (Zehra and Abbasi, 2018). These mutations altered enhancer activity through modified binding of key TFs of brain development, which in turn orchestrated the expression of genes involved in human brain development and function (Zehra and Abbasi, 2018; Khatoon et al., 2023; Li et al., 2023).

**Fig. 3.**
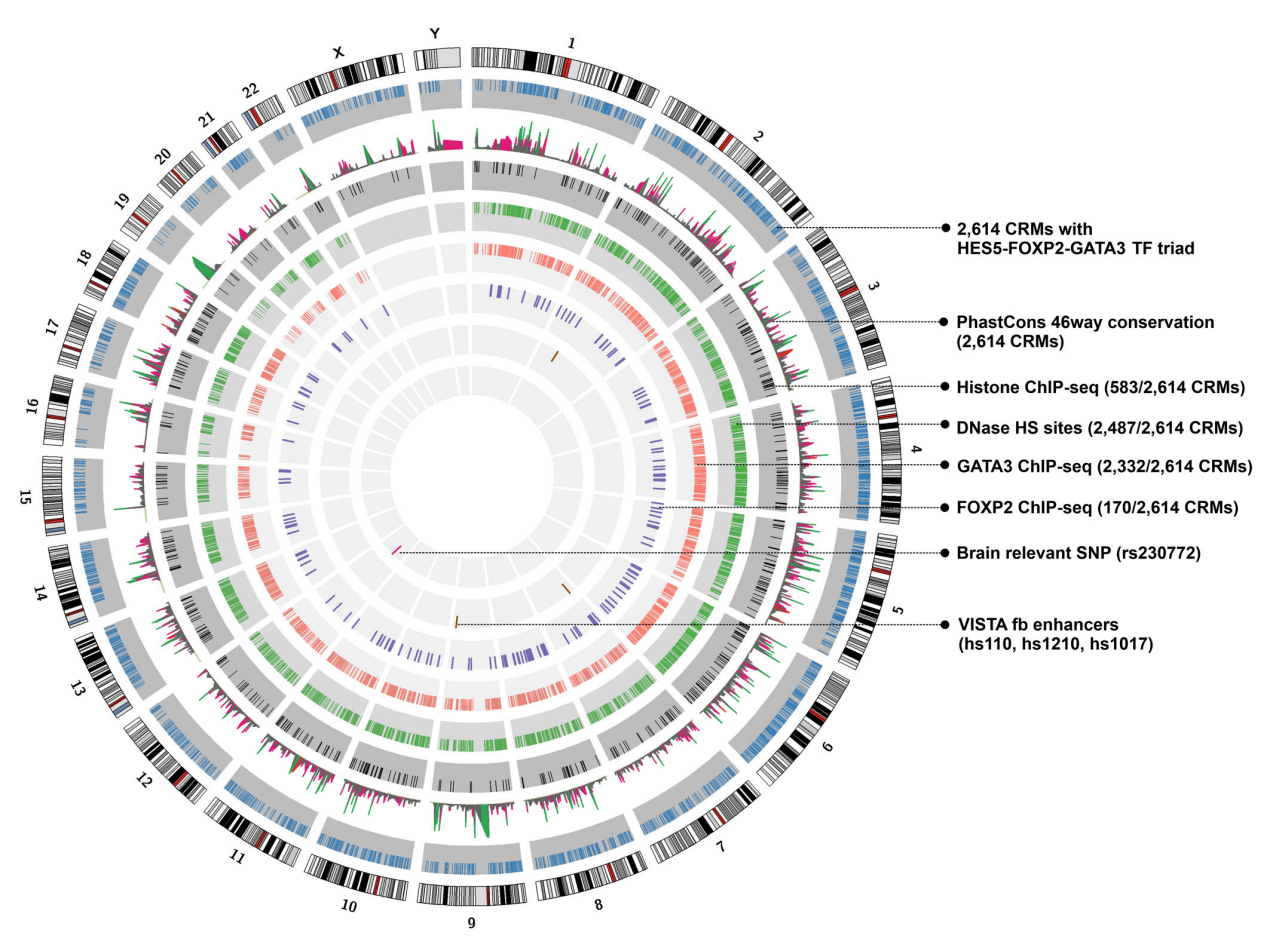
**Genome-wide distribution of 2614 forebrain CRMs that harbor the TF triad HES5-FOXP2-GATA3.** The circular panel shows a Circos image (generated by Circos software; https://circos.ca/) illustrating the human genome ideogram. Each chromosome is distinctly represented with specific banding patterns on the outermost layer. The second layer depicts the genome-wide distribution of 2614 human forebrain CRMs containing structured motif arrangements for the TF triad HES5-FOXP2-GATA3 (light blue tiles). The third layer graphically illustrates the conservation depths of these CRMs, represented here as bar plots where height and color of peaks represent the phastCons scores [grey peaks represent the primate clade (phastCons score <0.2), pink peaks represent the mammalian clade (phastCons score 0.2-0.5), red peaks represent birds (phastCons score 0.501-0.60), and green peaks represent amphibians and teleost fish (phastCons score >0.6)]. The fourth layer portrays a subset of CRMs (583/2614) that harbor activating histone modification marks such as H3K4me1 (based on human brain tissue from the ENCODE, https://www.encodeproject.org/) as well as H3K4me2 and H3K27ac (based on human embryonic cerebral cortex tissue data from the Gene Expression Omnibus, NCBI; Table S9) (black tiles). The fifth layer (green tiles) shows a subset of |CRMs (2487/2614) that harbor DNase HS sites derived from human brain cell lines data from ENCODE and human fetal brain tissues from Gene Expression Omnibus, NCBI (Table S10). The sixth and seventh layers depict the presence of |GATA3-specific (2332/2614; warm orange tiles) and FOXP2-specific (170/2614; purple tiles) ChIP-seq marks on subsets of CRMs (Tables S11,S12). The eighth layer shows functionally validated forebrain-specific enhancers from the VISTA Enhancer Browser (hs110, hs1210, and hs1017) that overlap with identified CRMs (brown tiles). The ninth and innermost layer shows GWAS-derived brain disease-associated SNP (rs2630772) whose coordinates overlap with forebrain CRM (chr12_crm_281) (magenta tiles). Fb, forebrain; CRM: *cis*-regulatory module; H3K4me1: histone H3 lysine 4 mono-methylation; H3K4me2: histone H3 lysine 4 di-methylation; H3K27ac: histone H3 lysine 27 acetylation; DNase HS, DNase hypersensitive sites; SNPs, single nucleotide polymorphisms; ENCODE, Encyclopedia of DNA Elements.

Given the significant phenotypic consequences of both evolutionarily conserved and lineage-specific enhancers, we investigated the evolutionary depth of 2614 forebrain CRMs containing the heterotypic TF triad: HES5-FOXP2-GATA3, using PhastCons 46way data from the UCSC Genome Browser (https://genome.ucsc.edu/). PhastCons, a hidden Markov model-based method, computes conservation scores ranging from 0 to 1, representing probabilities of negative selection. This method has been previously used to identify functionally relevant conserved non-coding regions (Siepel et al., 2005; Hubisz et al., 2010). PhastCons assigns conservation scores to these regions and categorizes them based on specific phastCons scores thresholds (Park et al., 2014). Using these thresholds in this analysis, it was found that most of the CRMs (2051/2614; ∼78%) harboring the TF triad pattern are conserved only among primates (phastCons score:<0.2) (Figs 2, 3; Table S7). Nearly 16% (414/2614) of the CRMs were conserved within the mammalian lineage, including rodents, canines, and afrotherians (phastCons score: 0.2-0.5) (Figs 2, 3; Table S7). Interestingly, only a small proportion of CRMs were conserved in non-mammalian vertebrates (phastCons score: >0.5). Specifically, 0.8% (21/2614) of the CRMs conserved in birds (*Gallus gallus*), and 4.5% (128/2614) of CRMs conserved in amphibians and teleost fish (*Xenopus tropicalis*, zebrafish) (Figs 2, 3; Table S7). These findings, highlighting the conservation depths of 2614 CRMs, signify the primate-specific functional relevance of the ordered placement of HES5, FOXP2, and GATA3 on forebrain enhancers.

### Epigenomic evidence of functional relevance for identified set of 2614 forebrain-specific CRMs

One of the defining characteristics of enhancer regions is their specific chromatin features, such as activating biochemical signatures like H3K4me1, H3K4me2, and H3K27ac (Creyghton et al., 2010; Pekowska et al., 2010; Kubo et al., 2024), their sensitivity to cleavage by the DNase I enzyme (DNase HS sites) (Song et al., 2011), and their tendency to bind to transcription factor proteins (Guo and Gifford, 2017). These chromatin marks are considered pivotal in predicting active enhancers in mammals (Lee et al., 2011). Given these well-defined chromatin-based features of active enhancers, we evaluated the regulatory potential of a genome-wide set of 2614 forebrain CRMs (containing the structured binding sites of HES5-FOXP2-GATA3) by intersecting them with H3K4me2 and H3K27ac marks from the, human embryonic cerebral cortex tissue, available in the Gene Expression Omnibus-NCBI and H3K4me1 marks data from human brain tissues available at ENCODE (Feingold et al., 2004; Clough and Barrett, 2016) (Tables S8 and S9); DNase I hypersensitive sites (DNase HS sites) from human brain cell lines and primary tissue samples derived from ENCODE and Gene Expression Omnibus-NCBI (Feingold et al., 2004; Clough and Barrett, 2016) (Tables S8 and S10). These intersections using BEDtools (v2.17.0) (Quinlan and Hall, 2010), revealed that 583/2614 forebrain CRMs overlapped with ChIP-seq data for H3K4me1, H3K4me2, and H3K27ac, while 2487/2614 CRMs overlapped with DNase HS sites (Figs 2, 3; Tables S9 and S10).

As an additional line of evidence, ENCODE-based human brain cell line-specific ChIP-seq datasets for transcription factors GATA3 (SK-N-SH cell line) and FOXP2 (SK-N-MC and PFSK-1 cell lines) were employed on the 2614 forebrain CRMs harboring the heterotypic TF triad: HES5-FOXP2-GATA3 (where FOXP2 binding sites is positioned between HES5 and GATA3 sites) (Feingold et al., 2004) (Table S8). The results showed that 2332/2614 forebrain CRMs overlapped with GATA3-specific ChIP-seq marks, while 170/2614 overlapped with FOXP2-specific ChIP-seq marks (Figs 2, 3; Tables S11 and S12). Taken together, a core set of 573 forebrain CRMs was identified that appears to harbor nearly all of the *cis*-regulatory epigenetic features analyzed in the present study. (Fig. 2; Table S15).

**Fig. 4.**
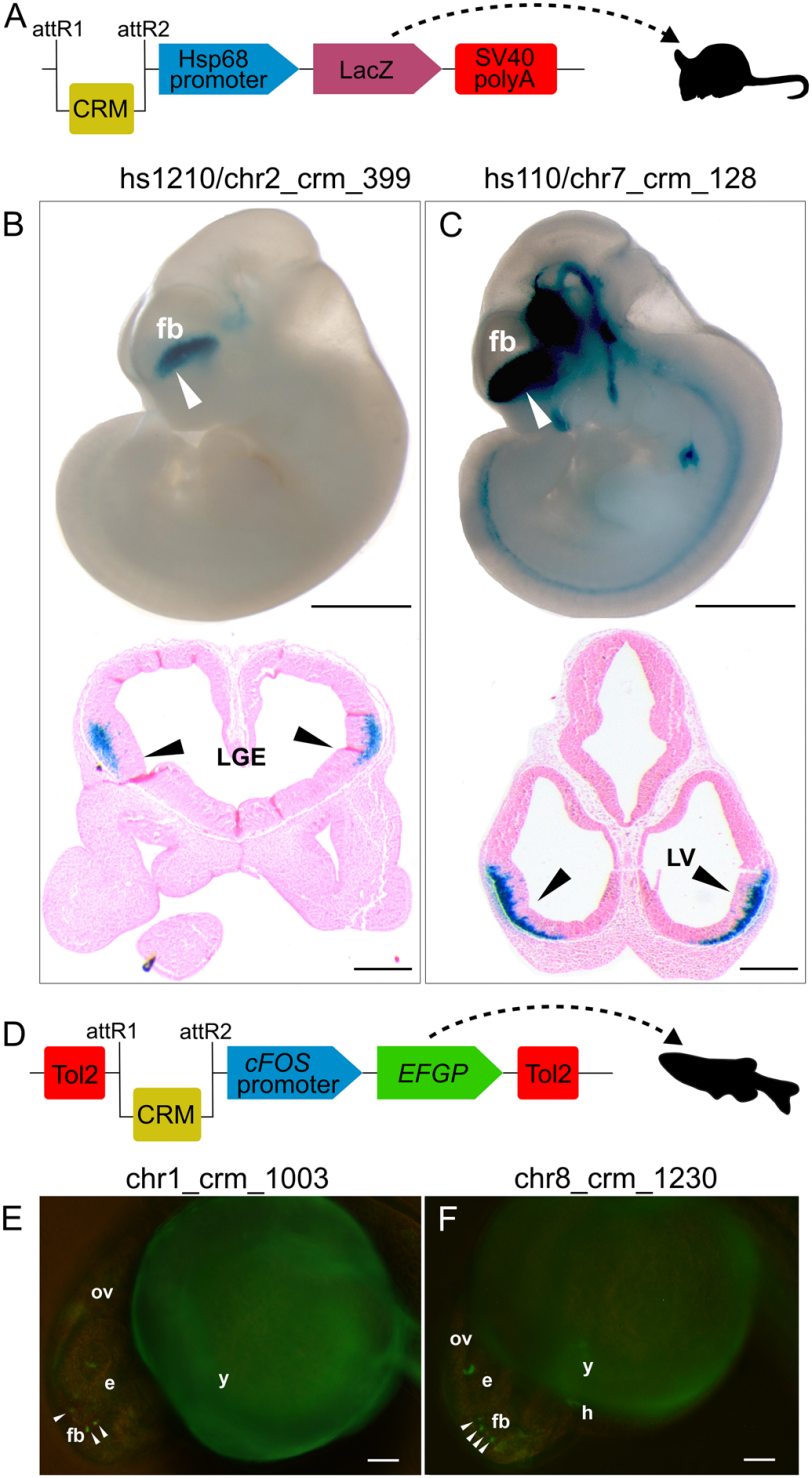
***In-vivo* functional relevance of identified forebrain-specific CRMs harboring the heterotypic TF triad HES5-FOXP2-GATA3.** This figure illustrates the regulatory activity domains of four representative CRMs (among a predicted genome-wide set of 2614 forebrain CRMs) containing the heterotypic TF triad HES5-FOXP2-GATA3, where the FOXP2 binding site is positioned between the HES5 and GATA3 sites. The figure includes reporter expression data for CRMs chr2_crm_399, chr7_crm_128, chr1_crm_1003, and chr8_crm_1230. The genomic coordinates of two of these predicted forebrain CRMs, chr2_crm_399 and chr7_crm_128, overlap with VISTA Enhancer Browser (https://enhancer.lbl.gov/) characterized forebrain-specific enhancers hs1210 and hs110, respectively. Therefore, the representative regulatory activity for these two CRMs (chr2_crm_399 and chr7_crm_128) depicted in B and C is derived from the VISTA enhancer browser. The forebrain-specific regulatory activity of the other two CRMs, chr1_crm_1003 and chr8_crm_1230, presented in E and F, was evaluated in the present study through transgenic analysis in zebrafish embryos. (A) The schematic shows reporter constructs carrying CRMs hs1210/chr2_crm_399 and hs110/chr7_crm_128, used to assay *cis*-regulatory activity in transgenic mice experiments. The vector backbone contains the Hsp68 (heat shock protein 68) promoter, LacZ reporter gene (β-galactosidase), and gateway recombination sites (attR1 and attR2). Detailed vector information is available in the VISTA Enhancer Browser (https://enhancer.lbl.gov/). (B,C) Whole-mount views of transgenic mouse embryos expressing the reporter under the control of hs1210/chr2_crm_399 and hs110/chr7_crm_128, respectively, at E11.5, along with transverse forebrain sections for each respective element. The white arrowheads in B and C indicate CRMs-induced lacZ expression in the forebrain (fb). Transverse sections through the forebrain revealed X-gal staining in the prospective lateral ganglionic eminence (LGE, black arrowhead) for hs1210/chr2_crm_399 and lateral ventricles (LV, black arrowhead) of the telencephalon for hs110/chr7_crm_128. (D) The schematic illustrates the reporter construct for carrying out *in-vivo* assays in transgenic zebrafish embryos. This Tol2 transposase expression vector [pGW_cfos-EGFP, (Fisher et al., 2006) contains the reporter gene EGFP (enhanced green fluorescent protein] and cfos promoter. The CRMs chr1_crm_1003 and chr8_crm_1230 were cloned in the gateway recombination sites (attR1 and attR2) upstream of the promoter. (E,F) These panels show the reporter gene (GFP) expression induced by CRMs chr1_crm_1003 and chr8_crm_1230 in the forebrain region of zebrafish embryos at approximately 24-48 h post-fertilization (hpf), as indicated by white arrowheads (zoomed-in images provided as panels A and B in Fig. S3). Note: in the case of transgenic zebrafish assays for CRMs chr1_crm_1003 and chr8_crm_1230, the reporter gene (GFP) expression was reproducible only in the forebrain, whereas reporter expression observed in other embryonic domains was not reproducible, probably due to mosaicism associated with this assay (percentage of embryos with GFP expression in forebrain, provided in Table S18). Following the generally accepted convention, zebrafish embryo images are shown with the anterior (head) to the left and dorsal to the top. For clarity, zebrafish embryonic domains are labeled as: (e) Eye; (y) yolk; (ov) otic vesicle; (h) heart; (fb) forebrain. Scale bars: B,C, E11.5 mouse embryo whole-mount views (1 mm) and respective transverse forebrain sections (500 µm); E,F, live zebrafish embryo (100 µm).

To further validate the functional relevance of 2614 CRMs, they were overlapped with functionally characterized *in-vivo* catalog of 320 mammalian forebrain enhancers from the VISTA Enhancer Browser (Visel et al., 2006) (Fig. 2; Table S13). This analysis revealed that three CRMs—chr7_crm_128, chr2_crm_399, and chr9_crm_642—overlapped with the VISTA forebrain enhancers hs110, hs1210, and hs1017, respectively (Figs 3 and 4A-C; Table S13). Moreover, given the role of regulatory DNA in human disease pathogenesis (Maurano et al., 2012; Welter et al., 2013), the 2614 CRMs were scanned for human brain-specific disease SNPs derived from the GWAS archive (Buniello et al., 2018). Notably, chr12_crm_281 harbored a SNP (rs2630772) associated with hippocampal volume atrophy (Mather et al., 2015) (Fig. 2, Table S14). To further assess the *in-vivo* functional relevance of these 2614 CRMs containing the heterotypic TF triad: HES5-FOXP2-GATA3, a subset of four CRMs, with significant epigenetic marks were selected and functionally evaluated using *in-vivo* transgenic zebrafish assay (Fig. 4D-F; Fig. S2, Table S16).

### *In-vivo* validation of identified forebrain *cis*-regulatory modules

To validate the *cis*-regulatory potential of forebrain CRMs containing the HES5-FOXP2-GATA3 triad, the Tol2 transposase assay was used for transgenesis in zebrafish, a model organism for studying human enhancers (Fisher et al., 2006; Parisi et al., 2021). This assay was conducted on a selected subset of four CRMs (chr2_crm_399, chr1_crm_1003, chr5_crm_1671 and chr8_crm_1230) (Table S16). These CRMs were selected based on the presence of important enhancer-associated epigenetic marks (Table S16). Three of these CRMs (chr2_crm_399, chr1_crm_1003 and chr5_crm_1671) had enhancer-associated epigenetic marks (Histone ChIP-seq marks, DNase HS sites and TF-ChIP-seq marks), while the fourth CRM, chr8_crm_1230, was devoid of histone ChIP-seq marks but harbored DNase HS sites and TF-ChIP-seq marks (Table S16). Chr2_crm_399 (human genome assembly GRCh37/hg19, chr2:66762756-66763557) overlapped with hs1210 from VISTA Enhancer Browser (Fig. 4B; Table S16) (https://enhancer.lbl.gov). This VISTA-documented forebrain-specific enhancer (hs1210) drove reporter gene expression exclusively in the developing forebrain of four out of five transgenic mouse embryos at E11.5 (Visel et al., 2006) (Fig. 4A,B). Consistent with the transgenic mouse data for hs1210, chr2_crm_399 induced reporter gene expression in the developing forebrain of approximately 50% of injected zebrafish embryos at 24 hpf (Figs S2, S3, Table S18). The conserved regulatory activity of chr2_crm_399 and hs1210 in the zebrafish and mouse forebrain, respectively, suggests the tissue specificity of these enhancers across diverse vertebrate species (Fig. 4B; Fig. S2). Similarly, reproducible GFP signal was detected in 43% of embryos injected with chr1_crm_1003 (Fig. 4D,E; Fig. S3, Table S18) and in 40% embryos injected with chr5_crm_1671 (Figs S2, S3, Table S18). Despite lacking histone ChIP-seq signatures, chr8_crm_1230 also induced reporter gene expression in the developing forebrain of 45% of injected zebrafish embryos (Fig. 4F; Fig. S3, Table S18).

These *in-vivo* data indicate that the absence of a particular cell line/tissue-specific epigenetic marks (histone modification marks, TF-ChIP-seq and DNase HS sites) does not necessarily rule out the functional relevance of these forebrain CRMs. The subset of CRMs lacking epigenetic marks, particularly those integrated into our functional evaluation scheme, might still harbor distinct histone modification marks and DNase HS sites and could be active in alternative cell types or developmental stage (Zentner et al., 2011; Pollex and Furlong, 2017). Moreover, the limited evolutionary conservation of the 2614 CRMs containing the HES-FOXP2-GATA3 motif suggests that tissue-specific grammatical code can help identify less well-conserved, lineage-specific regulatory elements that might be missed in comparative genomics studies of mammals and other vertebrates (Kuderna et al., 2024).

It is important to note that the scope of this study is focused on defining tissue-specific enhancer syntax through the ordered arrangement of a minimal set of TF binding motifs within non-coding genomic sequences. By selecting a minimal set of TF motifs and specifying their ordered placement, we aim to improve the specificity of enhancer prediction while also reducing the computational resources needed to scan the vast non-coding regions of human and other mammalian genomes, which comprise 98.5% of mammalian DNA.

Nevertheless, our study does not attempt to explore the mechanistic aspects of tissue-specific enhancer functionality, which are governed by several complex factors. These include co-binding of TFs to specific DNA sequences (Zanconato et al., 2015), the spacing between individual TF binding motifs (Rastegar et al., 2008), the presence of numerous heterotypically cooperative and homotypically clustered motifs (Georgakopoulos-Soares et al., 2023), TF-DNA binding energetics (Farrel et al., 2016), epigenomic modifications (Luo et al., 2022), and chromatin interaction dynamics (Liu et al., 2015).

### Conclusion

In the present study, we identified a tissue-specific combinatorial binding pattern of TFs, characterized by constraints in relative motif positioning but flexibility in binding site spacing. Our identified TF combinatorial binding model aligns with the spectrum of previously proposed schemes regarding the assembly of TF proteins on tissue-specific enhancers, such as the rigid ‘enhanceosome’ and the flexible ‘billboard’ organization. We successfully detected our devised TF combinatorial binding syntax for forebrain tissue in a significant fraction (2614 out of 25,000; ∼10.5%) of a previously reported genome-wide catalog of putative human forebrain enhancers. These 2614 putative forebrain enhancers, which harbor spatially co-occurring binding sites for HES5, FOXP2, and GATA3, can serve as important genomic templates for investigating forebrain-relevant gene regulatory networks and identifying their roles in forebrain-specific diseases, developmental processes, and evolutionary mechanisms. Furthermore, the tissue-specific TF combinatorial binding-based enhancer prediction model proposed in this study not only enhances our understanding of the functional anatomy of tissue-specific mammalian enhancers but also serves as a general framework for tissue-specific enhancer discovery. This model is particularly useful for identifying *cis*-regulatory regions without well-characterized epigenetic signals and those missed by traditional sequence conservation-based approaches.

A limitation of our work is that functional validation has been conducted for only four of the 2614 predicted forebrain CRMs featuring the HES5-FOXP2-GATA3 triad. To fully ascertain the relevance of this TF combination in forebrain-specific regulatory mechanisms, further *in vivo* or *in vitro* functional studies are required to validate additional CRMs containing this binding pattern.

## MATERIALS AND METHODS

### Identification of discriminatory motif grammar within an *in vivo* catalog of enhancers

To identify tissue-specific motif grammar, we retrieved a dataset of functionally confirmed FSHEs from the VISTA Enhancer Browser (Visel et al., 2006) (Table S1). As of March 2024, the VISTA Enhancer Browser contains information on an *in vivo* catalog of ∼320 human non-coding elements exhibiting gene enhancer activity in different brain regions of transgenic mice (https://enhancer.lbl.gov/). From these ∼320 enhancers, we selected a subset of 100 enhancers that showed endogenous expression profiles solely in the forebrain of transgenic mice (Table S1). To determine the transcription factor code potentially responsible for the forebrain specificity of these 100 VISTA enhancers, we adopted a set of 23 transcription factors (Shireen et al., 2025) (Table S2). These TFs express endogenously in mice brain/forebrain, are highly relevant to forebrain development and disease and have been reported as crucial for the forebrain specificity of human/mammalian non-coding *cis*-regulatory regions (Shireen et al., 2025) (Table S2). The list of 23 TFs was initially identified through a rigorous process involving an extensive literature review that confirmed their regulatory roles in human forebrain development (Zehra and Abbasi, 2018; Shireen et al., 2025) (Table S2). This was complemented by MGI (Mouse Genome Informatics; http://www.informatics.jax.org/) reported RNA *in situ* hybridization-based endogenous expression pattern investigation in the developing forebrain of mouse embryos, TF binding motif enrichment analysis, a phylogenetic footprinting-based TF motif conservation analysis, and statistical evaluation of their cooperative binding to forebrain enhancers (Shireen et al., 2025) (Fig. S1, Table S2). A flowchart detailing the various steps that led to the identification of this curated set of 23 TFs, highly relevant to the functionality of forebrain enhancers is summarized in Fig. S1, which is adapted from Shireen et al. (Shireen et al., 2025). The list of these 23 TFs, their TRANSFAC (release 2014.4) and JASPAR (release 2022) derived binding motifs (Wingender, 2008; Castro-Mondragon et al., 2021), and relevant literature citing their roles in forebrain development and disease, as well as their MGI based reported expression in brain domains is provided as Table S2.

Transcription Factor Binding Site Mapping Algorithm (TFBSMA; script available at the GitHub repository: https://github.com/HumaShireen/TFBSMA) was employed to search for the TRANSFAC/JASPAR derived binding motifs for 23 forebrain relevant TFs within the 100 FSHEs from VISTA Enhancer Browser (positive control dataset, Table S1) as well as 100 non-coding non-conserved sequences, NCNCSs (negative control dataset, Table S3). We then employed an association rule data mining approach, typically used in MBA (Raeder and Chawla, 2011) to identify the distinct and over-represented motif combinations among the 23 TFs frequently occurring in FSHEs (positive control) and NCNCSs (negative control). MBA has been adapted in previous studies to predict combinatorial transcription factors binding and the grammar of binding sites in human genome (Morgan et al., 2007; Bentsen et al., 2022). In our adapted MBA approach, we analyzed the positive (100 FSHEs) and negative (100 NCNCSs) control datasets using a Python implementation of the Apriori algorithm (Agrawal and Srikant, 1994) with a minimum support threshold of 0.1 and a minimum confidence threshold of 0.6 (available at: https://github.com/Fatiima-Batool/Enhancer-Combinatorial-Syntax/tree/main/MBA_Python). The co-occurrence of binding motifs for the 23 TFs was evaluated and compared between FSHEs (positive control) and NCNCSs (negative control) using key metrics: support, confidence, and lift (Table S4).

- Support (A→B) measures the joint probability of two or more factors co-occurring, i.e. Support (A∩B) is the proportion of enhancers where TFs A and B appear together.
- Confidence assesses the conditional probability that one TF appears when another is present.

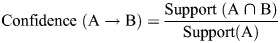
- Lift quantifies the strength of an association by determining how much more likely two or more TFs co-occur than if they were independent. A lift value >1 indicates a positive correlation (Raeder and Chawla, 2011).

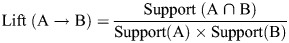

Multiple association rules with significant support and confidence values were identified for co-occurrence of TF binding motifs in pairs, as well as in combinations of three or four distinct TFs in FSHEs (positive control) (Table S4). To define the minimal forebrain-specific regulatory code, we selected the association rule involving at least three distinct TFs with the highest support and confidence (Fig. 1A; Table S4). Manual screening of the TF binding matrix of 23 transcription factors across positive control dataset identified 16 FSHEs where the binding motifs for HES5, FOXP2, and GATA3 were directly adjacent, with no intervening motifs (Table S1). Notably, 10/16 FSHEs with this TF triad contained FOXP2 binding site positioned between the HES5 and GATA3 sites (HES5-FOXP2-GATA3) (Fig. 1B). This motif combination was thus considered the TF motif-based syntax determining forebrain specificity (Fig. 1B; Table S1). The spacing between adjacent TF binding sites for TF triad HES5-FOXP2-GATA3 was also evaluated (Table S5).

The specific motif combination of HES5-FOXP2-GATA3 was then searched by using a Python script (source code available on GitHub: https://github.com/Fatiima-Batool/Enhancer-Combinatorial-Syntax/blob/main/Code.py) within a previously predicted genome-wide set of approximately 25,000 forebrain CRMs, recently deposited by our group at the Open Source repository platform DATADRYAD (Shireen et al., 2025) (https://datadryad.org/stash/share/LpDZxNHctzQGPr8AmBHwT8FAQOTkQohet7nBO2DlNe0). This Python script- based search resulted in the identification of TF motif combination HES5-FOXP2-GATA3 in 2614/25,000 of forebrain CRMs (Table S6).

### Analysis of conservation depth and validation of CRMs containing the heterotypic TF triad: HES5-FOXP2-GATA3

Sequence conservation of the 2614 forebrain CRMs, containing the heterotypic TF triad: HES5-FOXP2-GATA3 (with FOXP2 binding site positioned between HES5 and GATA3 sites), was estimated through phastCons (Siepel et al., 2005). The phastCons 46way data, generated using multiple sequence alignments of 46 vertebrate species was downloaded from the UCSC Genome Browser (http://hgdownload.cse.ucsc.edu/goldenpath/hg19/phastCons46way/). From this data, average phastCons scores were extracted for each of the 2614 forebrain CRMs (Table S7). Next, the thresholds were defined for categorizing the CRMs based on their average phastCons scores (Park et al., 2014) (Table S7). Three different thresholds were defined as follows:

- Less well conserved CRMs (primate-specific): average phastCons <0.2.
- Mammalian conserved CRMs: 0.2≤average phastCons≤0.5.
- Non-mammalian vertebrates conserved CRMs: average phastCons >0.5.

The 2614 categorized CRMs, along with their corresponding phastCons scores indicating conservation across primate, mammalian, or non-mammalian vertebrate clades, are visualized as conservation peaks in a Circos plot generated using Circos software (Fig. 3) (Krzywinski et al., 2009).

To assess the biological relevance of the shortlisted 2614 CRMs containing the forebrain-specific heterotypic TF triad: HES5-FOXP2-GATA3, their genomic coordinates were intersected with multiple datasets of epigenetic signatures using BEDtools (v2.17.0) (Quinlan and Hall, 2010). These datasets included: (1) activating histone modification marks from human embryonic cerebral cortex, specifically histone H3 lysine 4 di-methylation (H3K4me2) and histone H3 lysine 27 acetylation (H3K27ac), sourced from the Gene Expression Omnibus-NCBI (http://www.ncbi.nlm.nih.gov/geo/) as well as histone H3 lysine 4 mono-methylation (H3K4me1) marks datasets from human brain primary tissue available at ENCODE (Encyclopedia of DNA Elements, http://www.encodeproject.org/) (Feingold et al., 2004; Reilly et al., 2015; Clough and Barrett, 2016) (accession details for these datasets are provided in Table S8, with overlapping CRMs listed in Table S9); (2) DNase I hypersensitive sites (DNase HS sites), key markers of regulatory DNA, from human brain-specific cell lines and fetal brain tissue, sourced from ENCODE and Gene Expression Omnibus -NCBI (Feingold et al., 2004; Clough and Barrett, 2016) (accession details provided in Table S8; overlapping CRMs in Table S10); (3) ENCODE-based transcription factor-specific ChIP-seq datasets of GATA3 (SK-N-SH cell line) and FOXP2 (SK-N-MC and PFSK-1 cell lines) (Feingold et al., 2004) (Tables S8, S11,S12); (4) 320 *in-vivo* characterized forebrain relevant enhancers available at VISTA Enhancer Browser (overlapping CRMs listed in Table S13) (Visel et al., 2006). (5) Forebrain-specific archive of 4286 SNPs from GWAS (Buniello et al., 2018) (Table S14). Based on these intersections, a core dataset consisting of 573/2614 forebrain CRMs was defined, which contained multiple distinct epigenetic features: including activating histone modification marks H3K4me1, H3K4me2 and H3K27ac, DNase HS sites, as well as ChIP-seq marks for TFs GATA3 and FOXP2 (Table S15).

### Zebrafish transgenic assay

Among the genome-wide set of human 2614 forebrain CRMs that harbor the heterotypic TF triad: HES5-FOXP2-GATA3, a subset of four CRMs (chr2_crm_399, chr1_crm_1003, chr5_crm_1671, chr8_crm_1230) was selected for *in-vivo* analysis in transgenic zebrafish assay (Fig. 2; Table S16). Reporter constructs were prepared by cloning the corresponding segments of PCR amplified human CRMs (primer details in Table S17) into pCR8/GW/TOPO (entry vector) following manufacturer instructions (Invitrogen, Life Technologies) (https://tools.thermofisher.com/content/sfs/manuals/pcr8gwtopo_man.pdf). The confirmed cloned inserts were then shifted to pGW-cfos-EGFP (destination vector) through gateway cloning technology (Katzen, 2007). Using LR clonase enzyme; LR (attL and attR) recombination reaction between entry and destination clone; each 150 ng µl^−1^ was performed (Ali et al., 2021). The destination clones were confirmed by Sanger sequencing and restriction digestion. For microinjection, transposase mRNA was synthesized through sp6 mMessage mMachine kit (Ambion). Microinjection solution was prepared by following the protocol devised by Fisher et al., 2006 (Fisher et al., 2006).

Animal studies were approved by the Institutional Bioethics/Biosafety Committee, Office of the Dean, Faculty of Biological Sciences, Quaid-I-Azam University, Islamabad, Pakistan (no. DFBS/20-6094). In compliance with the NIH (National Research Council) guide for the care and use of laboratory animals, the AB strain of wild-type zebrafish were maintained and bred, and embryos were collected and staged as described (Kimmel et al., 1995; Westerfield, 2007). Approximately 2 nL of injection mixture was injected into the animal pole of 60-70 fertilized embryo at 1-2 cells stage. Microinjection for a minimum of three independent biological replicates was performed for each CRM. Following microinjection, at 24-48 h post fertilization (hpf), embryos were dechorionated and anesthetized with tricaine methanesulfonate (MS-222) and observed under a fluorescent inverted microscope IX71 (Olympus, Japan) to detect reporter gene expression (Green fluorescent protein, GFP). Fluorescent photomicrographs were obtained at 24-48 hpf. All four CRMs drove reproducible reporter gene expression in the developing forebrain of transgenic zebrafish embryos at 24 hpf. However, the GFP signals observed in tissues other than the forebrain were non-reproducible and could be attributed to the mosaic nature of this assay (Fig. 2). A CRM is generally considered to have enhancer activity in a given tissue if at least 20% of injected zebrafish embryos exhibit reproducible reporter gene expression in that tissue (Abrar et al., 2024) (Table S18).

### Declaration of ethical guidelines for studies in animals

The *in-vivo* assay in zebrafish reported in this study complies with the ARRIVE (Animal Research: Reporting of *In Vivo* Experiments) guidelines and adheres to the NIH Guide for the Care and Use of Laboratory Animals, with fulfillment of Article 33 of EU Directive 2010/63 for animal experiments.

In compliance with the NIH guide for the care and use of laboratory animals, AB strain of wild-type zebrafish was maintained in optimal water quality conditions and housed at appropriate densities to reduce stress. The temperature and dark/light cycle were maintained. Fish were provided with a balanced diet, and their health was regularly monitored. Quarantine procedures were implemented when necessary to prevent disease spread. Breeding of adult male and female zebrafish (3-6 months old) was conducted in suitable conditions within specialized spawning tanks, and fertilized eggs were promptly collected to avoid predation by adult fish. Repeated inbreeding was avoided to maintain genetic diversity. Microinjection was performed on 60-70 fertilized eggs at the 1-2 cell stage, and the larvae were reared under appropriate water parameters and nutritional conditions. Transgenic zebrafish embryos (24-48 h post-fertilization) were humanely anesthetized for fluorescent microscopy to observe GFP (green fluorescent protein) signals in the developing forebrain.

## Supplementary Material

10.1242/biolopen.061751_sup1 Supplementary information

Table S1. Prediction of binding sites of 23 forebrain-relevant TFs (listed in Table S2) on in-vivo characterized 100 human forebrain-specific cis-regulatory elements acquired from the VISTA Enhancer Browser.

Table S2. List of 23 transcription factors expressed endogenously in forebrain and are relevant to human/mammalian forebrain disease and development.

Table S3. Prediction of binding sites of 23 forebrain-relevant TFs (listed in Table S1) on 100 non-coding, non-conserved sequences (NCNCSs).

Table S4. MBA-based (Market Basket Analysis) support, confidence and lift values of association rules observed for distinct binding motif combinations among 23 TFs, identified through association rule data mining of the datasets of 100 forebrain specific human enhancers (FSHEs) and 100 non-coding non-conserved sequences (NCNCSs).

Table S5. Distance in base pairs between adjacent motifs in the heterotypic module comprising the TF triad HES5-FOXP2-GATA3

Table S6. A genome-wide list of 2,614 forebrain-specific CRMs containing the heterotypic TF triad: HES5-FOXP2-GATA3 (where FOXP2 binding site is positioned between HES5 and GATA3 sites).

Table S7. The identified set of 2,614 forebrain-specific CRMs harboring the heterotypic TF triad: HES5-FOXP2-GATA3 (where FOXP2 binding site is positioned between HES5 and GATA3 sites), subjected to conservation depth analysis using the University of California Santa Cruz (UCSC) Genome Browser based phastCons 46way (https://genome.ucsc.edu/).

Table S8. Details of datasets of human brain-specific DNase-I hypersensitive sites, Histone ChiP-seq (H3K4me1, H3K4me2, and H3K27ac) and TF Chip-Seq marks (GATA3, FOXP2) derived from ENCODE (Encyclopedia of DNA Elements: https://www.encodeproject.org/) and Gene Expression Omnibus-NCBI (https://www.ncbi.nlm.nih.gov/geo/).

Table S9. Identified set of 2,614 CRMs harboring the heterotypic TF triad: HES5-FOXP2-GATA3 (where FOXP2 binding site is positioned between HES5 and GATA3 sites), subjected to functional validation using activating histone modifications marks; H3K4me1 from human fetal brain derived from ENCODE (Encyclopedia of DNA Elements, https://www.encodeproject.org/); H3K4me2 and H3K27ac from the human cerebral cortex, derived from Gene Expression Omnibus-NCBI (https://www.ncbi.nlm.nih.gov/geo/).

Table S10. Identified set of 2,614 CRMs harboring the heterotypic TF triad: HES5-FOXP2-GATA3 (where FOXP2 binding site is positioned between HES5 and GATA3 sites), subjected to functional validation using DNase-I hypersensitive sites from human brain cell lines and primary tissue samples derived from ENCODE (Encyclopedia of DNA Elements; https://www.encodeproject.org/) and Gene Expression Omnibus-NCBI (https://www.ncbi.nlm.nih.gov/geo/).

Table S11.This table shows that out of 2,614 forebrain-specific CRMs (containing the heterotypic TF triad: HES5-FOXP2-GATA3, where FOXP2 binding site is positioned between HES5 and GATA3 binding sites), 2,332 CRMs were enriched with GATA3-specific ChIP-seq marks for human brain cell line (SK-N-SH) derived from ENCODE [(Encyclopedia of DNA Elements; https://www.encodeproject.org/) (ENCODE accession: ENCSR000BTH)]. Column 2 lists the human chromosome number, columns 3 and 4 show the start and end coordinate positions (human genome assembly GRCh37/hg19) of CRMs overlapping with GATA3-ChIP-seq marks, and column 5 contains the unique identifier for each overlapped CRM.

Table S12. Identified set of 2,614 CRMs harboring the heterotypic TF triad: HES5-FOXP2-GATA3 (where FOXP2 binding sites is positioned between HES5 and GATA3 sites), subjected to functional relevance investigation using ENCODE (Encyclopedia of DNA Elements) based ChIP-seq marks for transcription factor FOXP2 from human brain cell lines (SK-N-MC and PFSK-1) (https://www.encodeproject.org/).

Table S13. Identified set of 2,614 CRMs harboring the heterotypic TF triad: HES5-FOXP2-GATA3 (where FOXP2 binding sites is positioned between HES5 and GATA3 sites), subjected to functional validation using forebrain relevant enhancer functionality of 320 mammalian forebrain enhancers from VISTA Enhancer Browser (https://enhancer.lbl.gov/)

Table S14. Identified set of 2,614 CRMs harboring the heterotypic TF triad: HES5-FOXP2-GATA3 (where FOXP2 binding sites is positioned between HES5 and GATA3 sites), assessed for disease relevance using human brain-specific SNPs from the Genome Wide Association Studies (GWAS) catalog (https://www.ebi.ac.uk/gwas/).

Table S15. The list of 573 forebrain-specific CRMs containing the heterotypic TF triad: HES5-FOXP2-GATA3 (where FOXP2 binding site is positioned between HES5 and GATA3 sites) and displaying enrichment for atleast three different cis-regulatory features such as epigenetic marks, forebrain relevant enhancer functionality and disease relevance.

Table S16. A selected subset of 2,614 CRMs containing the heterotypic TF triad: HES5-FOXP2-GATA3 (where FOXP2 binding site is sandwiched between HES5 and GATA3 sites), subjected to in-vivo functional validation using transgenic zebrafish assays.

Table S17. Set of forward and reverse primers used for the PCR-based amplification of selected subset of forebrain-specific CRMs

Table S18. Annotation of tissue-specific activities from zebrafish transgenic reporter assays conducted for a selected subset of forebrain-specific CRMs.
