# Supplementary material for: The combinatorial binding syntax of transcription factors in forebrain-specific enhancers
Source: Biol Open. 2025 Feb 19;14(2):BIO061751. doi: 10.1242/bio.061751 (PMC11876843; doi:10.1242/bio.061751)
Supplement: Supplementary information [file biolopen-14-061751-s1.pdf]

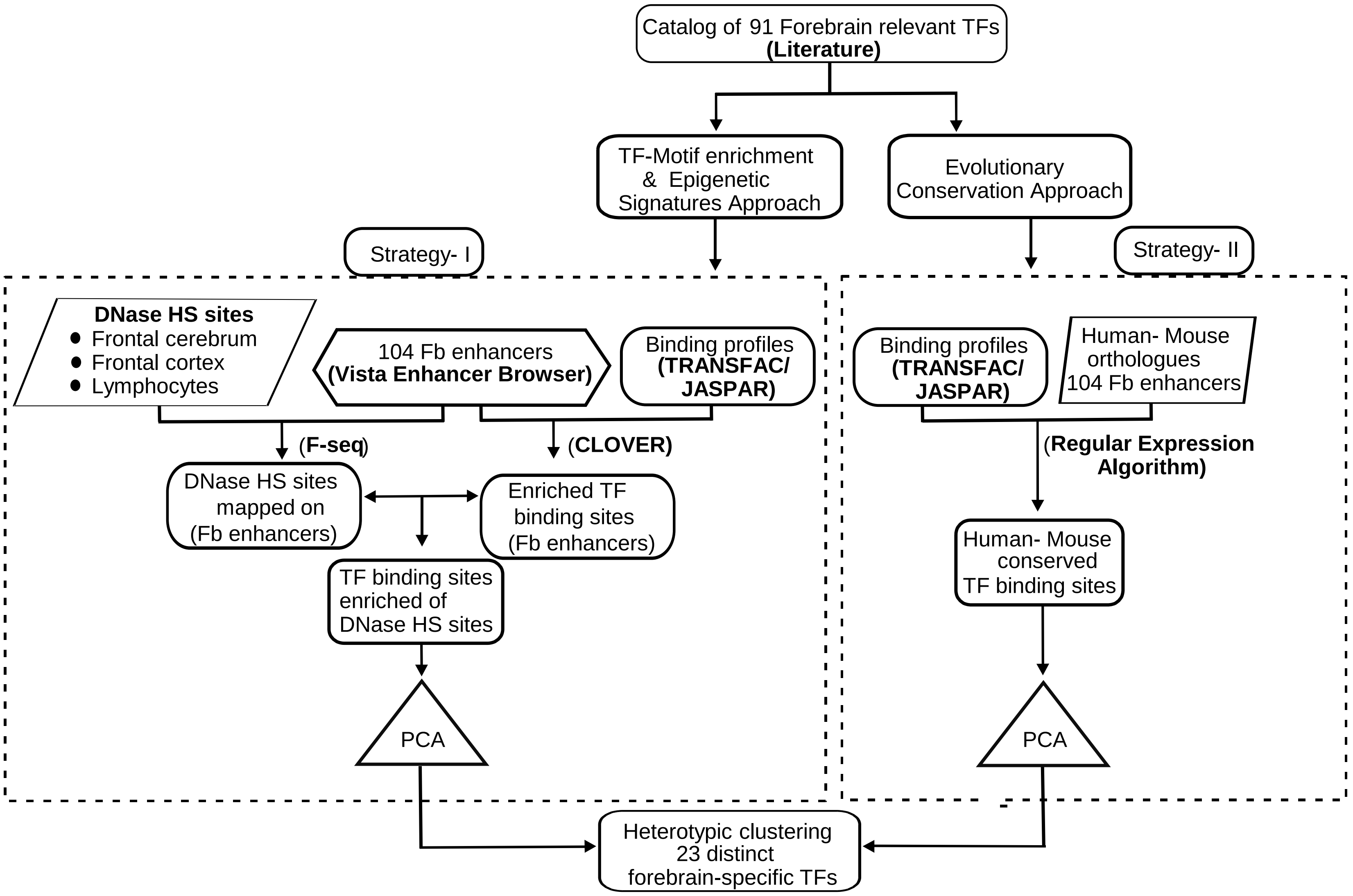

Fig. S1. This figure outlines two strategies (Strategy I and Strategy II) employed to characterize forebrain-specific heterotypic cooperativity among transcription factors (TFs) (Shireen et al., 2024)

<u>**Strategy I** involves the following steps:</u>

1. **Literature Review and Cataloging**: A set of 91 TFs relevant to mammalian forebrain development and disease was cataloged, focusing on their reported RNA in situ hybridization-based expression patterns in developing mouse forebrain (Zehra and Abbasi, 2018).
2. **TF Motif Enrichment:** Position weight matrices (PWMs) for the 91 TFs, derived from JASPAR (Castro-Mondragon et al., 2021) and TRANSFAC (Wingender, 2008), were subjected to motif enrichment analysis on a dataset of 104 human forebrain-specific enhancers (FSHEs) from the VISTA Enhancer Browser (Visel et al., 2006) using the Clover program (Frith et al., 2004).
3. **DNase I Hypersensitive Sites detection**: The F-seq algorithm (Song et al., 2011) was employed with ENCODE-based DNase I hypersensitive sites data (DNase HS sites) of GM12878 (B-lymphocyte), Cerebrum_Frontal_OC (frontal cerebrum tissue), and Frontal_Cortex_OC (ventromedial prefrontal cortex) (Feingold et al., 2004) to identify DNase HS sites in 104 FSHEs.
4. **Data Intersection**: F-seq predicted DNase HS sites on 104 FSHEs were intersected with Clover-predicted TF binding motifs, shortlisting TFBSs enriched in forebrain-specific human enhancers and associated with DNase I hypersensitive sites.

Biology Open • Supplementary information

Strategy II involves:

1. **Conserved Binding Sites Identification**: A regular expression-based algorithm (available on GitHub: https://github.com/HumaShireen/Regular-Expression-based-Algorithm) was employed to align human and mouse orthologous sequences, identifying human-mouse conserved binding sites for the 91 forebrain-related TFs in 104 FSHEs.

Analysis and Results:

- **Principal Component Analysis (PCA)**: The results of both strategies were transformed into TF occurrence-based frequency matrices (FMs) for 104 FSHEs and subjected to PCA for dimensionality reduction and identification of clusters of co-occurring TFs (Hand, 2007). PCA was performed using the R programming language, generating 3D loading plots showing distinct clusters of co-occurring TFs in the FSHEs.
- **TF Patterns Intersection**: Intersecting TF patterns from the 3D loading plots of both strategies resulted in the shortlisting of a minimum set of 23 distinct TFs that potentially bind cooperatively to forebrain enhancers.

Note: All steps summarized in Strategies I and II were also applied in parallel on a dataset of 100 non-coding, non-conserved sequences (NCNCSs, not shown in this flowchart) dispersed across the human genome, with an average length of ~1000 base pairs (Suppl. Table S3). The results for 100 NCNCSs were also subjected to PCA, which revealed no heterotypic interactive patterns of TFs in NCNCSs. This indicates that the heterotypic clustering of 23 TFs observed in 104 FSHEs is specific to forebrain development. Details of the 23 TFs are provided in Table S2.

Biology Open • Supplementary information

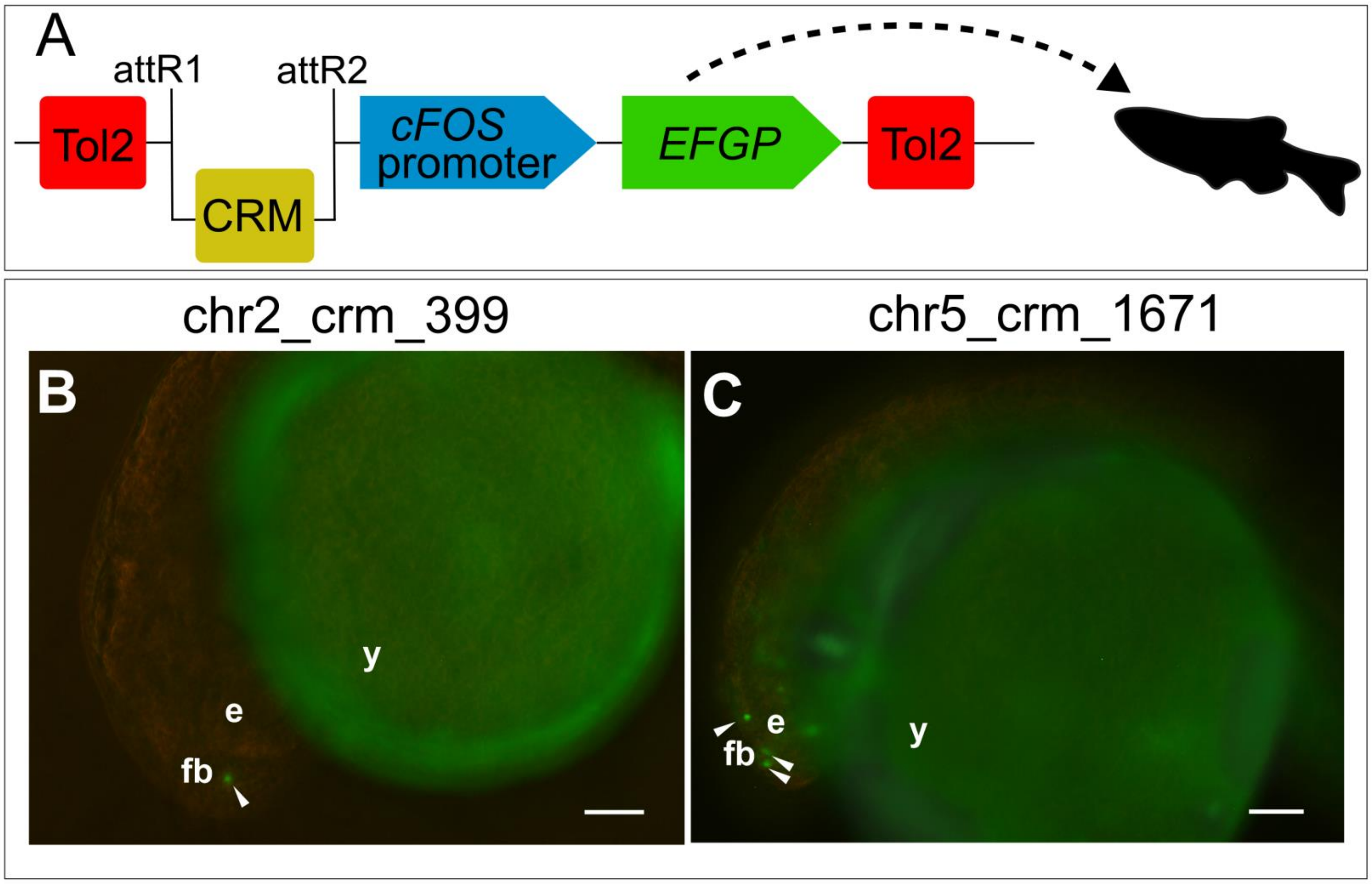

Fig. S2. Evaluation of functional relevance of identified forebrain-specific CRMs harboring the heterotypic TF: triad HES5-FOXP2-GATA3 in transgenic zebrafish assay.

Panel A: The schematic illustrates the reporter construct for carrying out in-vivo assays in transgenic zebrafish embryos. This Tol2 transposon expression vector (pGW_cfos-EGFP) (Fisher et al., 2006) contains the reporter gene EGFP (enhanced green fluorescent protein) and cfos promoter. The CRMs chr2_crm_399 and chr5_crm_1671 were cloned in the gateway recombination sites (attR1 and attR2) upstream of the promoter.

Panels B and C: These panels show the reporter gene (GFP) expression induced by CRMs chr2_crm_399 and chr5_crm_1671 in the forebrain region of zebrafish embryos at approximately 24-48 hours post-fertilization (hpf), as indicated by white arrowheads (zoomed in images provided as panels C-D in Fig. S3). **Note**: In the case of transgenic zebrafish assays for CRMs chr2_crm_399 and chr5_crm_1671, the reporter gene (GFP) expression was reproducible only in the forebrain, whereas reporter expression observed in other embryonic domains was not reproducible, probably due to mosaicism associated with this assay (percentage of embryos with GFP expression in forebrain, provided in Suppl. Table S18). Following the generally accepted convention, zebrafish embryo images are shown with the anterior (head) to the left and dorsal to the top. For clarity, zebrafish embryonic domains are labeled as: (e) Eye; (y) yolk; (fb) forebrain. Scale bars: B-C, 100 µm. The reporter expression data for other two CRMs that are evaluated for in vivo analysis in transgenic zebrafish assay is presented in Panels E and F of main Figure 4. Selection criteria and genomic features of subset of these elements selected for in vivo analysis in transgenic zebrafish assay are provided in Table S16.

Biology Open • Supplementary information

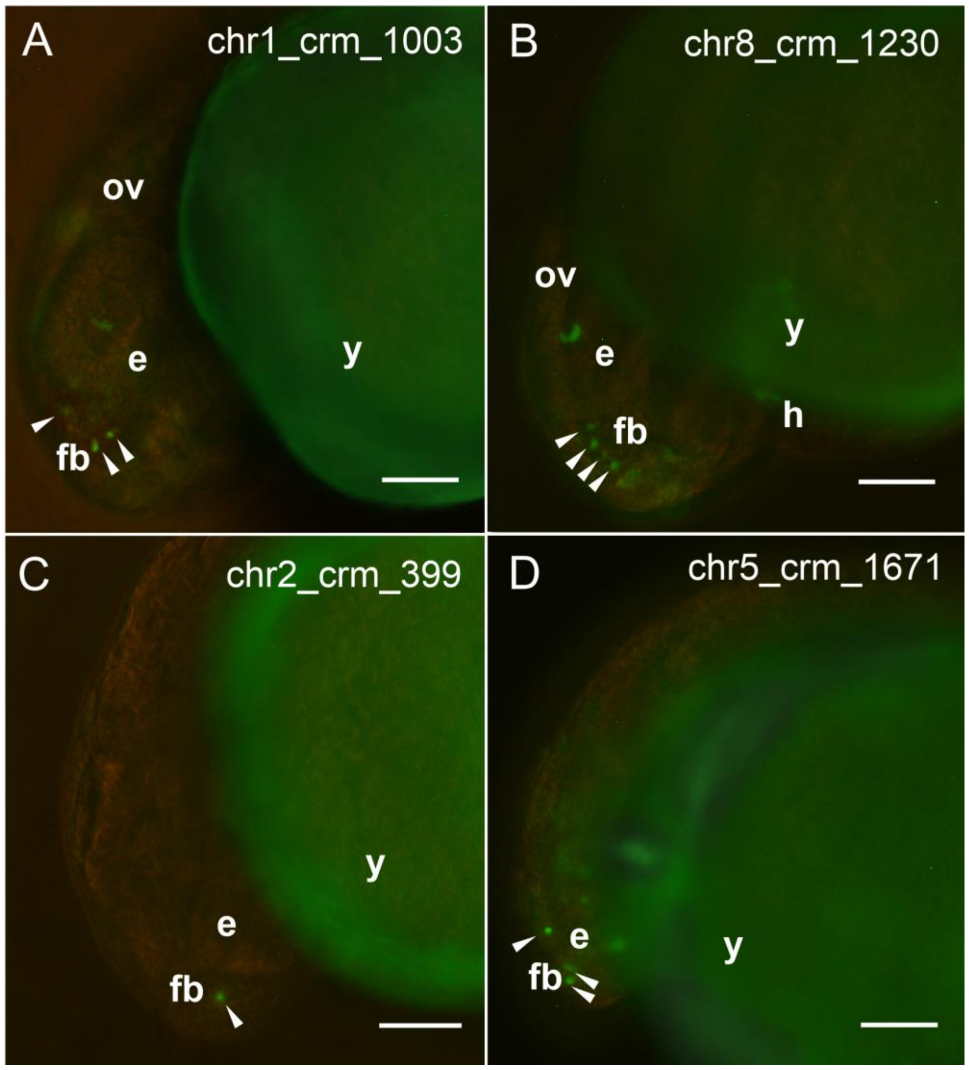

Fig. S3. Zoomed-in images of live zebrafish embryos showing GFP expression in forebrain derived by forebrain-specific CRMs harboring the heterotypic TF: triad HES5-FOXP2-GATA3

Panels A-D: These panels show the zoomed in view of reporter gene (GFP) expression induced by CRMs chr1_crm_1003 (Fig. 4E), chr8_crm_1230 (Fig. 4F), chr2_crm_399 (Fig. S2-B) and chr5_crm_1671 (Fig. S2-C) in the forebrain region of zebrafish embryos at approximately 24-48 hours post-fertilization (hpf), as indicated by white arrowheads. Zebrafish embryo images are shown with the anterior (head) to the left and dorsal to the top. For clarity, zebrafish embryonic domains are labeled as: (e) Eye; (y) yolk; (fb) forebrain; (h) heart; (ov) otic vesicle. Scale bars: A-D, 100 µm.

Biology Open • Supplementary information

Table S1. Prediction of binding sites of 23 forebrain-relevant TFs (listed in Table S2) on in-vivo characterized 100 human forebrain-specific cis-regulatory elements acquired from the VISTA Enhancer Browser.

Available for download at
https://journals.biologists.com/bio/article-lookup/doi/10.1242/bio.061751#supplementary-data

Table S2. List of 23 transcription factors expressed endogenously in forebrain and are relevant to human/mammalian forebrain disease and development.

Available for download at
https://journals.biologists.com/bio/article-lookup/doi/10.1242/bio.061751#supplementary-data

Table S3. Prediction of binding sites of 23 forebrain-relevant TFs (listed in Table S1) on 100 non-coding, non-conserved sequences (NCNCSs).

Available for download at
https://journals.biologists.com/bio/article-lookup/doi/10.1242/bio.061751#supplementary-data

Table S4. MBA-based (Market Basket Analysis) support, confidence and lift values of association rules observed for distinct binding motif combinations among 23 TFs, identified through association rule data mining of the datasets of 100 forebrain specific human enhancers (FSHEs) and 100 non-coding non-conserved sequences (NCNCSs).

Available for download at
https://journals.biologists.com/bio/article-lookup/doi/10.1242/bio.061751#supplementary-data

Table S5. Distance in base pairs between adjacent motifs in the heterotypic module comprising the TF triad HES5-FOXP2-GATA3

Available for download at
https://journals.biologists.com/bio/article-lookup/doi/10.1242/bio.061751#supplementary-data

Biology Open • Supplementary information

Table S6. A genome-wide list of 2,614 forebrain-specific CRMs containing the heterotypic TF triad: HES5-FOXP2-GATA3 (where FOXP2 binding site is positioned between HES5 and GATA3 sites).

Available for download at
https://journals.biologists.com/bio/article-lookup/doi/10.1242/bio.061751#supplementary-data

Table S7. The identified set of 2,614 forebrain-specific CRMs harboring the heterotypic TF triad: HES5-FOXP2-GATA3 (where FOXP2 binding site is positioned between HES5 and GATA3 sites), subjected to conservation depth analysis using the University of California Santa Cruz (UCSC) Genome Browser based phastCons 46way
(https://genome.ucsc.edu/).

Available for download at
https://journals.biologists.com/bio/article-lookup/doi/10.1242/bio.061751#supplementary-data

Table S8. Details of datasets of human brain-specific DNase-I hypersensitive sites, Histone ChiP-seq (H3K4me1, H3K4me2, and H3K27ac) and TF Chip-Seq marks (GATA3, FOXP2) derived from ENCODE (Encyclopedia of DNA Elements: https://www.encodeproject.org/) and Gene Expression Omnibus-NCBI (https://www.ncbi.nlm.nih.gov/geo/).

Available for download at
https://journals.biologists.com/bio/article-lookup/doi/10.1242/bio.061751#supplementary-data

Table S9. Identified set of 2,614 CRMs harboring the heterotypic TF triad: HES5-FOXP2-GATA3 (where FOXP2 binding site is positioned between HES5 and GATA3 sites), subjected to functional validation using activating histone modifications marks; H3K4me1 from human fetal brain derived from ENCODE (Encyclopedia of DNA Elements, https://www.encodeproject.org/); H3K4me2 and H3K27ac from the human cerebral cortex, derived from Gene Expression Omnibus-NCBI (https://www.ncbi.nlm.nih.gov/geo/).

Available for download at
https://journals.biologists.com/bio/article-lookup/doi/10.1242/bio.061751#supplementary-data

Table S10. Identified set of 2,614 CRMs harboring the heterotypic TF triad: HES5-FOXP2-GATA3 (where FOXP2 binding site is positioned between HES5 and GATA3 sites), subjected to functional validation using DNase-I hypersensitive sites from human brain cell lines and primary tissue samples derived from ENCODE (Encyclopedia of DNA Elements; https://www.encodeproject.org/) and Gene Expression Omnibus-NCBI (https://www.ncbi.nlm.nih.gov/geo/).

Available for download at
https://journals.biologists.com/bio/article-lookup/doi/10.1242/bio.061751#supplementary-data

Table S11. This table shows that out of 2,614 forebrain-specific CRMs (containing the heterotypic TF triad: HES5-FOXP2-GATA3, where FOXP2 binding site is positioned between HES5 and GATA3 binding sites), 2,332 CRMs were enriched with GATA3-specific ChIP-seq marks for human brain cell line (SK-N-SH) derived from ENCODE [(Encyclopedia of DNA Elements; https://www.encodeproject.org/) (ENCODE accession: ENCSR000BTH)]. Column 2 lists the human chromosome number, columns 3 and 4 show the start and end coordinate positions (human genome assembly GRCh37/hg19) of CRMs overlapping with GATA3-ChIP-seq marks, and column 5 contains the unique identifier for each overlapped CRM.

Available for download at
https://journals.biologists.com/bio/article-lookup/doi/10.1242/bio.061751#supplementary-data

Table S12. Identified set of 2,614 CRMs harboring the heterotypic TF triad: HES5-FOXP2-GATA3 (where FOXP2 binding sites is positioned between HES5 and GATA3 sites), subjected to functional relevance investigation using ENCODE (Encyclopedia of DNA Elements) based ChIP-seq marks for transcription factor FOXP2 from human brain cell lines (SK-N-MC and PFSK-1) (https://www.encodeproject.org/).

Available for download at
https://journals.biologists.com/bio/article-lookup/doi/10.1242/bio.061751#supplementary-data

Table S13. Identified set of 2,614 CRMs harboring the heterotypic TF triad: HES5-FOXP2-GATA3 (where FOXP2 binding sites is positioned between HES5 and GATA3 sites), subjected to functional validation using forebrain relevant enhancer functionality of 320 mammalian forebrain enhancers from VISTA Enhancer Browser (https://enhancer.lbl.gov/)

Available for download at
https://journals.biologists.com/bio/article-lookup/doi/10.1242/bio.061751#supplementary-data

Table S14. Identified set of 2,614 CRMs harboring the heterotypic TF triad: HES5-FOXP2-GATA3 (where FOXP2 binding sites is positioned between HES5 and GATA3 sites), assessed for disease relevance using human brain-specific SNPs from the Genome Wide Association Studies (GWAS) catalog (https://www.ebi.ac.uk/gwas/).

Available for download at
https://journals.biologists.com/bio/article-lookup/doi/10.1242/bio.061751#supplementary-data

Table S15. The list of 573 forebrain-specific CRMs containing the heterotypic TF triad: HES5-FOXP2-GATA3 (where FOXP2 binding site is positioned between HES5 and GATA3 sites) and displaying enrichment for atleast three different cis-regulatory features such as epigenetic marks, forebrain relevant enhancer functionality and disease relevance.

Available for download at
https://journals.biologists.com/bio/article-lookup/doi/10.1242/bio.061751#supplementary-data

Table S16. A selected subset of 2,614 CRMs containing the heterotypic TF triad: HES5-FOXP2-GATA3 (where FOXP2 binding site is sandwiched between HES5 and GATA3 sites), subjected to in-vivo functional validation using transgenic zebrafish assays.

Available for download at
https://journals.biologists.com/bio/article-lookup/doi/10.1242/bio.061751#supplementary-data

Table S17. Set of forward and reverse primers used for the PCR-based amplification of selected subset of forebrain-specific CRMs

Available for download at
https://journals.biologists.com/bio/article-lookup/doi/10.1242/bio.061751#supplementary-data

Table S18. Annotation of tissue-specific activities from zebrafish transgenic reporter assays conducted for a selected subset of forebrain-specific CRMs.

Available for download at
https://journals.biologists.com/bio/article-lookup/doi/10.1242/bio.061751#supplementary-data

References for supplementary information

Shireen, H., Batool, F., Khatoon, H., Parveen, N., Sehar, N. U., Hussain, I., Ali, S. and Abbasi, A. A. (2024). Predicting genome-wide tissue-specific enhancers via combinatorial transcription factor genomic occupancy analysis. *FEBS Lett.* https://10.1002/1873-3468.15030

Zehra, R. and Abbasi, A. A. (2018). Homo sapiens-specific binding site variants within brain exclusive enhancers are subject to accelerated divergence across human population. *Genome Biol. Evol.* **10,** 956-966. https://doi.org/10.1093/gbe/evy052

Castro-Mondragon, J. A., Riudavets-Puig, R., Rauluseviciute, I., Berhanu Lemma, R., Turchi, L., Blanc-Mathieu, R., Lucas, J., Boddie, P., Khan, A., Manosalva Pérez, N., et al. (2021). JASPAR 2022: the 9th release of the open-access database of transcription factor binding profiles. *Nucleic Acids Res.* **50,** D165-D173. https://doi.org/10.1093/nar/gkab1113

Wingender, E. (2008). The TRANSFAC project as an example of framework technology that supports the analysis of genomic regulation. *Brief. Bioinform.* **9,** 326-332. https://doi.org/10.1093/bib/bbn016

Visel, A., Minovitsky, S., Dubchak, I. and Pennacchio, L. A. (2006). VISTA Enhancer Browser—a database of tissue-specific human enhancers. *Nucleic Acids Res.* **35,** D88-D92. https://doi.org/10.1093/nar/gkl822

Frith, M. C., Fu, Y., Yu, L., Chen, J. F., Hansen, U. and Weng, Z. (2004). Detection of functional DNA motifs via statistical over-representation. *Nucleic Acids Res.* **32,** 1372-1381. https://doi.org/10.1093/nar/gkh299

Song, L., Zhang, Z., Grasfeder, L. L., Boyle, A. P., Giresi, P. G., Lee, B. K., Sheffield, N. C., Gräf, S., Huss, M., Keefe, D., et al. (2011). Open chromatin defined by DNaseI and FAIRE identifies regulatory elements that shape cell-type identity. *Genome Res.* **21,** 1757-1767. https://doi.org/10.1101/gr.121541.111

Feingold, E., Good, P., Guyer, M., Kamholz, S., Liefer, L., Wetterstrand, K., Collins, F., Gingeras, T., Kampa, D., Sekinger, E., et al. (2004). The ENCODE (ENCyclopedia Of DNA Elements) Project. *Science.* **306,** 636-640. https://doi.org/10.1126/science.1105136

Hand, D. J. (2007). Principles of Data Mining. *Drug Saf.* **30,** 621-622. https://doi.org/10.2165/00002018-200730070-00010

Fisher, S., Grice, E. A., Vinton, R. M., Bessling, S. L., Urasaki, A., Kawakami, K. and McCallion, A. S. (2006). Evaluating the biological relevance of putative enhancers using Tol2 transposon-mediated transgenesis in zebrafish. *Nat. Protoc.* **1,** 1297-1305. https://doi.org/10.1038/nprot.2006.230

Biology Open • Supplementary information
